# Supplementary material for: Lanthanide luminescence nanothermometer with working wavelength beyond 1500 nm for cerebrovascular temperature imaging in vivo
Source: Nat Commun. 2024 Mar 15;15:2341. doi: 10.1038/s41467-024-46727-5 (PMC10943110; doi:10.1038/s41467-024-46727-5)
Supplement: Supplementary file 1 — Supplementary Information [file 41467_2024_46727_MOESM1_ESM.pdf]

Supplementary Information

Lanthanide luminescence nanothermometer with working wavelength beyond 1500 nm for cerebrovascular temperature imaging in vivo

Yukai Wu[1,5], Fang Li[1,5], Yanan Wu[2], Hao Wang[1], Liangtao Gu[2], Jieying Zhang[1], Yukun Qi[2], Lingkai Meng[1], Na Kong[1], Yingjie Chai[3], Qian Hu[1], Zhenyu Xing[1], Wuwei Ren[2], Fuyou Li[3,4]* and Xingjun Zhu[1]**

[1]School of Physical Science and Technology, ShanghaiTech University, 393 Middle Huaxia Road, Shanghai 201210, P.R. China.

[2]School of Information Science and Technology, ShanghaiTech University, 393 Middle Huaxia Road, Shanghai 201210, P.R. China.

[3]Department of Chemistry & State Key Laboratory of Molecular Engineering of Polymers & Collaborative Innovation Center of Chemistry for Energy Materials, Fudan University, 2005 Songhu Road, Shanghai 200438, P.R. China.

[4]Institute of Translational Medicine, Shanghai Jiao Tong University, 800 Dongchuan Road, Shanghai, 200240, P.R. China.

[5]These authors contributed equally: Yukai Wu, Fang Li.

*e-mail: renww@shanghaitech.edu.cn; fyli@fudan.edu.cn; zhuxj1@shanghaitech.edu.cn

Material: Erbium chloride ($ErCl_3$), Yttrium chloride (YCl_3) and Ytterbium ($YbCl_3$) all were homemade. Oleic acid (OA, technical grade, 90 %) and 1-octadecene (ODE, technical grade, 90 %) were purchased from Sigma-Aldrich. N,N-Dimethylformamide (DMF, AR, ≥ 99.5 %), ethanol (AR, ≥ 99.7 %), Dichloromethane (DCM, AR ≥ 99.5 %) and methanol (AR, ≥ 99.5 %) were purchased from Greagent. Ammonium fluoride (NH_4F, AR ≥ 98.0 %) and cyclohexane (99.9 %) were bought from Adamas. Nitrosonium tetrafluoroborate ($NOBF_4$) was purchased from Alfa Aesar. 1, 2-distearoyl-sn-glycero-3-phosphoethanolamine-poly(ethylene glycol) (DSPE-PEG) was purchased from J&K. Sodium hydroxide (NaOH, AR, ≥ 96.0 %) and gelatin (CP) was purchased from Sinopharm. Lipopolysaccharide (LPS) was purchased from Absin Bioscience Inc. Dimethyl-sulfoxide-D6 (DMSO, ≥ 99.9 %) was purchased from Solarbio. Phosphate buffer saline (PBS) was purchased from Beyotime. Hemoglobin was purchased from Aladdin. The cell medium was made of Dulbecco's Modified Eagle Medium (DMEM, Mediatech, Inc.), 10 % (v/v) of fetal bovine serum (FBS, Beyotime) and 1 % (v/v) antibiotics (streptomycin and penicillin). All the chemicals were utilized without further purification.

Characterizations: The morphology and size of the core and core/shell nanoparticles were characterized by transmission electron microscopy (TEM) using a JEM-1400plus microscope at an acceleration voltage of 120 kV. Size histograms were calculated from TEM images after analyzing the dimensions of at least 100 particles using the Nano Measure software. X-ray powder diffraction (XRD) patterns of as-synthesized nanoparticles were measured on a BRUKER D2 PHASER DESKTOP XRD using Cu Kα radiation (λ= 0.15418 nm) at a scanning speed of 5 °/min. The NIR luminescence spectra of all nanoparticles were obtained using a near IR spectrometer (NIR17S spectrometer, Ideaoptics, China) with an 850 nm long pass filter (FELH0850, Thorlabs) in front of the detector, at excitation of 808 nm laser. The luminescence spectra in VIS region of all NPs were obtained using a FX2000 fiber spectrometer (Ideaoptics, China) with a 760 nm short-pass filter in front of the detector, at excitation of 808 nm laser. All the spectra under varied temperature was obtained by putting the cuvette into the temperature controller (Qpod 2e, QUANTUM NORTHWEST). The luminescence

lifetimes of Er^{3+} ions at its $^4I_{11/2}$ and $^4I_{13/2}$ excited state were characterized on a time-resolved photoluminescence spectrometer (FLS-1000, Edinburgh) using the 808 nm pulsed laser. Fourier transform infrared (FT-IR) spectra were measured using a FT-IR Spectrometer (Spectrum Two, PerkinElmer). Dynamic light scattering of DSPE-PEG modified nanoparticles in aqueous environment were performed on a Zetasizer Nano (Malvern Instruments Ltd., UK). The optical density (OD) of each well in cytotoxicity assay was detected using imaging reader (Cytation 5, BioTek). The NIR-II/III *in vivo* luminescence images were recorded by NIR-II/III *in vivo* imaging system (Monet IGS-1000P, Suzhou NIR-Optics Co., Ltd., China). The images at 980 nm channel and 1550 nm channel were recorded under cooperation of 900 nm long-pass (FEL900, Thorlabs) and 1400 nm long-pass filters (FEL1400, Thorlabs) respectively. Water absorption spectra were tested through UV-VIS-NIR spectrophotometer (Carry 5000, Agilent).

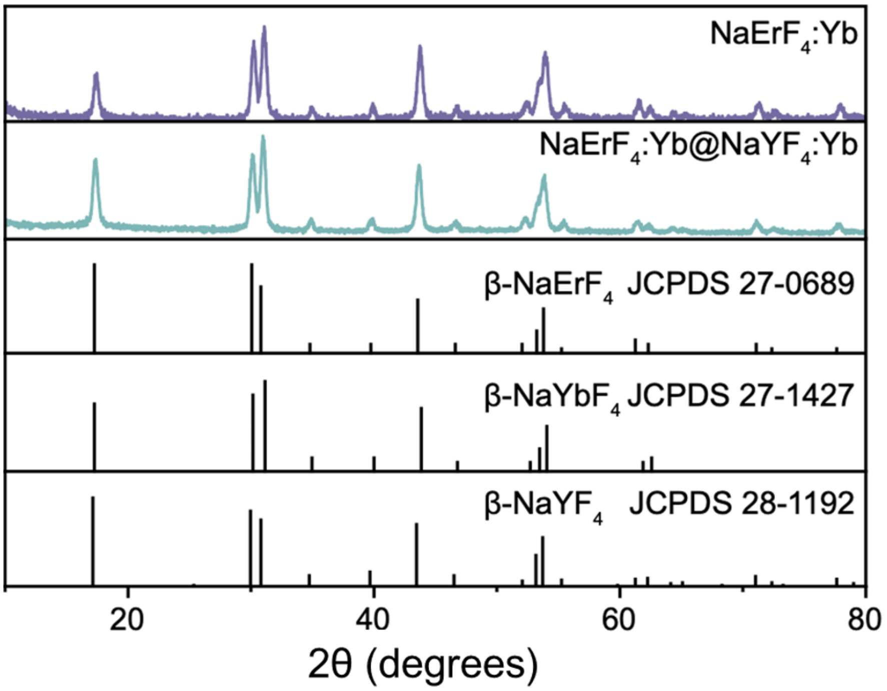

Supplementary Figure 1 | Crystal phase characterization. X-ray powder diffraction (XRD) analysis of $NaErF_4$:Yb and $NaErF_4$:Yb@$NaYF_4$:Yb. The typical XRD patterns of hexagonal phases of β-$NaErF_4$ (standard JCPDS file No. 27-0689), β-$NaYbF_4$

(standard JCPDS file No. 27-1427) and β-$NaYF_4$ (standard JCPDS file No. 28-1192) were given.

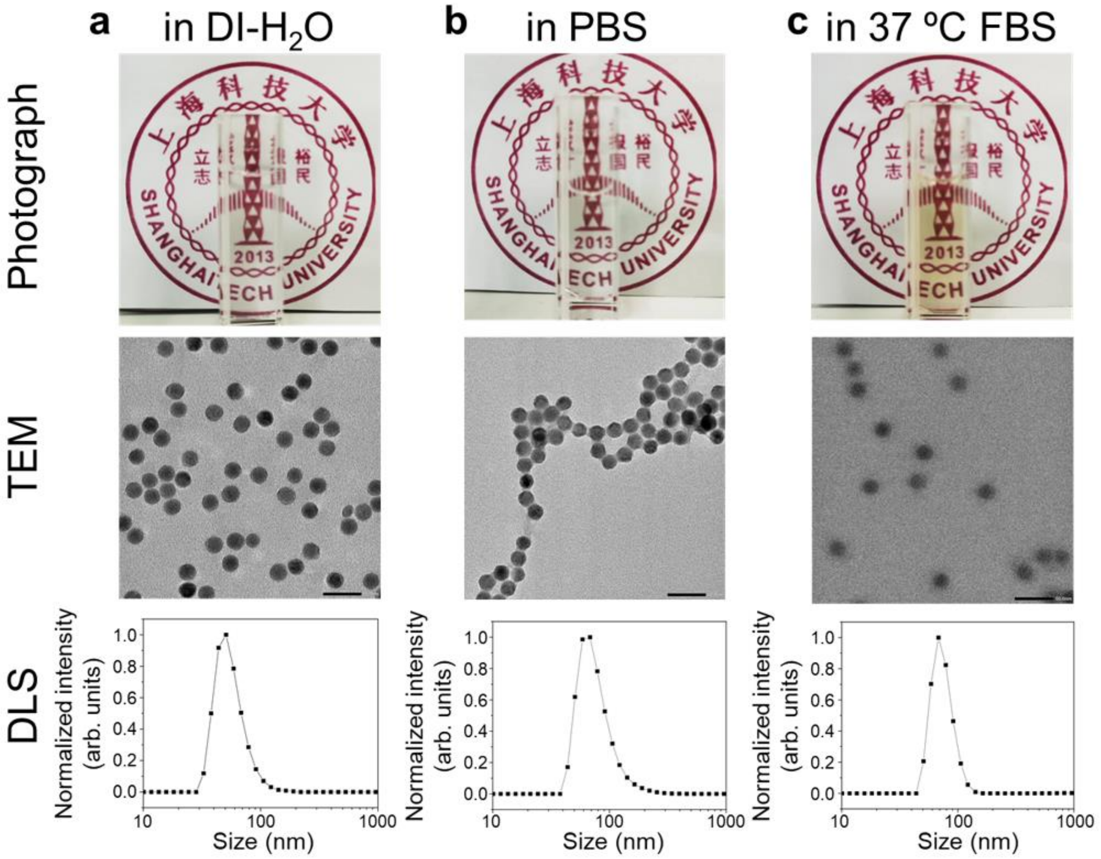

Supplementary Figure 2 | Dispersity of LIBRA in various aqueous solutions. Photographs (upper row), transmission electron microscopy (TEM) images (middle row) and dynamic light scattering (DLS) measurements (lower row) of LIBRA dispersed in (a) deionized water (DI-H_2O), (b) phosphate buffer saline (PBS) and (c) fetal bovine serum (FBS) solution at 37 ºC. TEM images represent the similar results of experiments repeated independently for three times. Scale bars in TEM images are 50 nm.

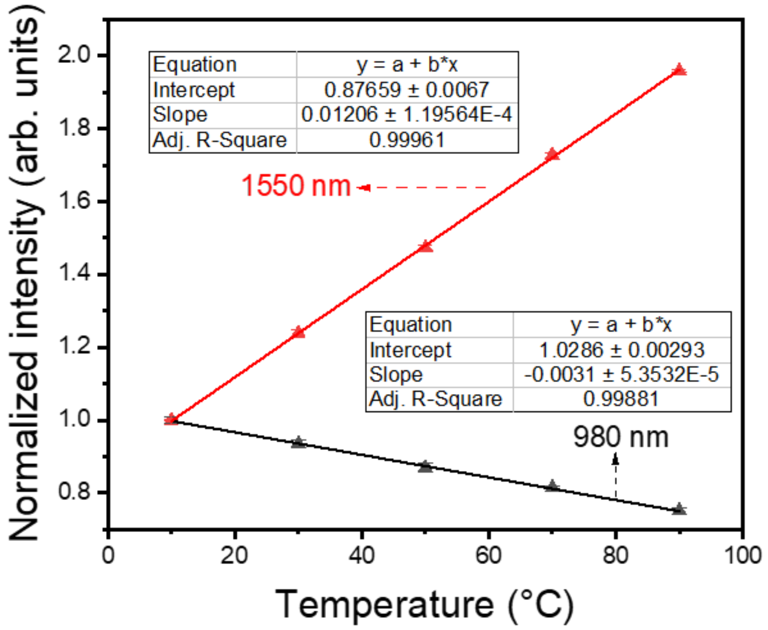

Supplementary Figure 3 | Temperature responsive behavior of emissions of LIBRA. Normalized intensity and linear fit of 980 nm and 1550 nm emissions of LIBRA under various temperature. The intensities were normalized at 10 °C to compare the slopes of fitted curves of different emissions. Data were given in mean intensity values based on three measurements (n = 3) of the luminescence spectra. Error bars were defined as standard deviation.

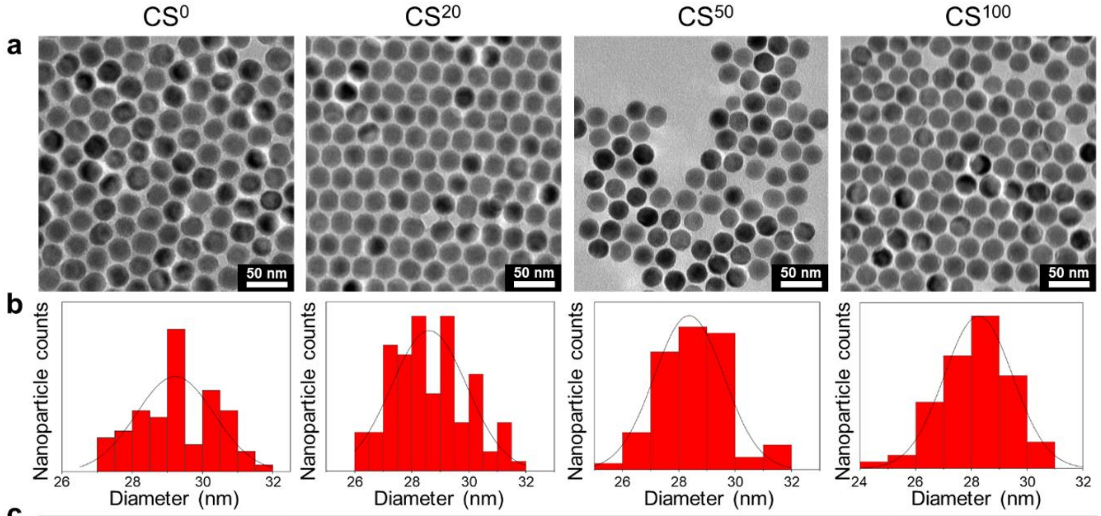

c

x % Yb in active shell	0	20	50	100
Size (nm)	29.22 ± 1.11	28.61 ± 1.32	28.36 ± 1.24	28.25 ± 1.23
Shell thickness (nm)	3.70	3.65	3.52	3.47

Supplementary Figure 4 | Morphology characterization of LIBRA with different Yb^{3+} doping ratios in the shell. (a) Transmission electron microscopy (TEM) images of LIBRA with various concentration of Yb^{3+} in the shell (0, 20, 50 and 100 %, respectively, denoted as CS^{0}, CS^{20}, CS^{50} and CS^{100}, respectively). TEM images represent the similar results of experiments repeated independently for three times. (b) Size distribution diagrams of CS^{0}, CS^{20}, CS^{50} and CS^{100}. (c) Statistic particle sizes and shell thicknesses of CS^{0}, CS^{20}, CS^{50} and CS^{100}.

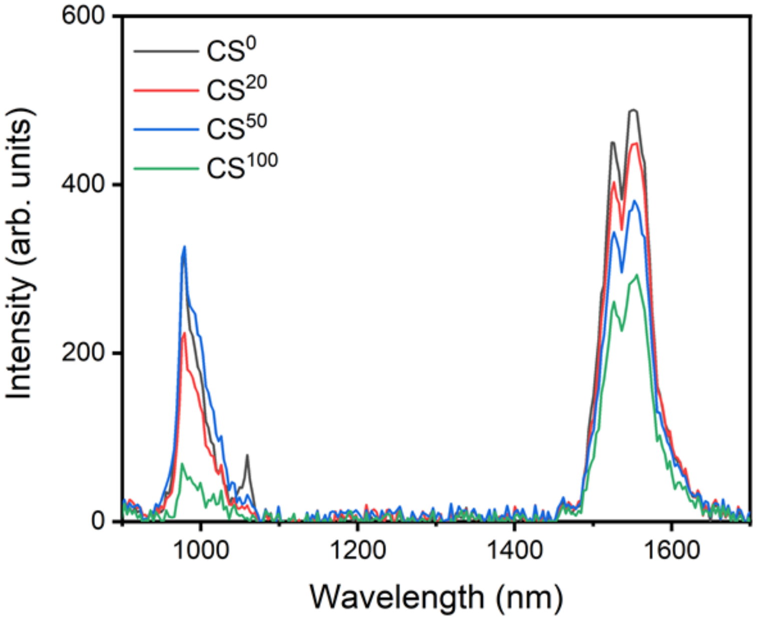

Supplementary Figure 5 | Luminescence property of LIBRA. Luminescence spectra of LIBRA ($NaErF_4$:20%Yb@$NaYF_4$:Yb) with varied concentrations of Yb^{3+} in shell layer (0, 20, 50 and 100 %, respectively, which are denoted as CS^0, CS^{20}, CS^{50} and CS^{100}, respectively) in aqueous solution.

a

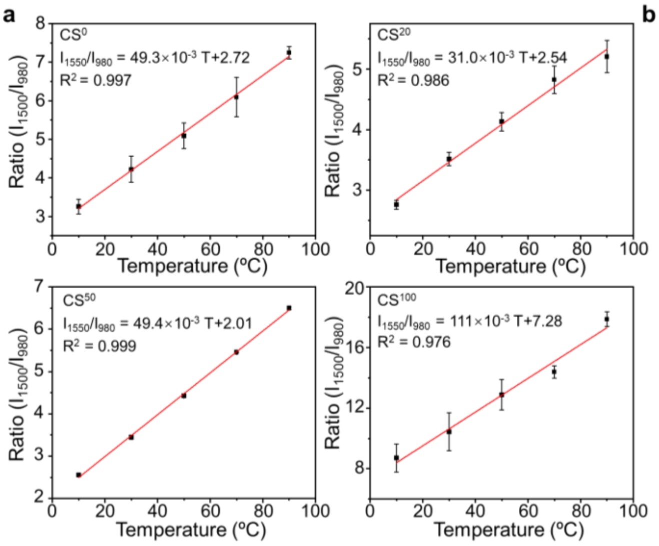

b

	S_r I_{1550}/I_{980} at 310 K (% K^{-1})
0 % Yb	1.09
20 % Yb	0.84
50 % Yb	1.29
100 % Yb	0.98

Supplementary Figure 6 | Temperature responsive behavior of LIBRA with different Yb^{3+} doping ratios in the shell layer. (a) The ratio of emissions at 1550 and 980 nm (I_{1550}/I_{980}) *versus* temperature (T) for CS^0, CS^{20}, CS^{50} and CS^{100} nanoparticles in aqueous environment. Data were presented as mean ratio values based on three measurements (n = 3) of the emission spectra. Error bars were defined as standard

deviation. (b) Corresponding relative thermal sensitivity (S_r) at 310 K for I_{1550}/I_{980} *versus* T.

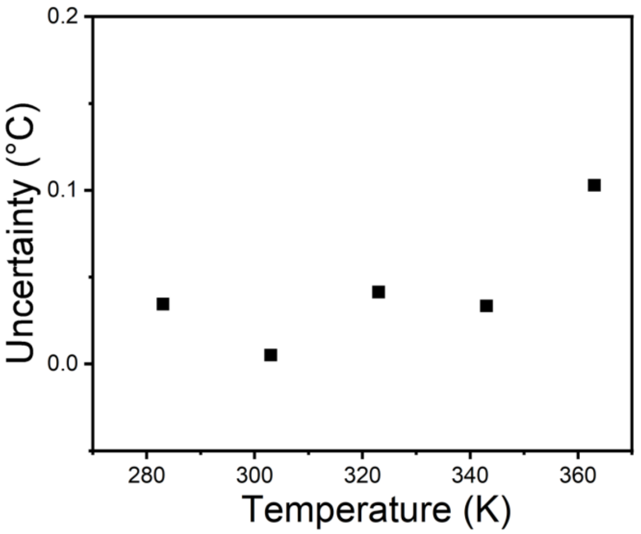

Supplementary Figure 7 | Temperature detection performance of LIBRA. Temperature uncertainty of LIBRA under different temperatures in aqueous solution.

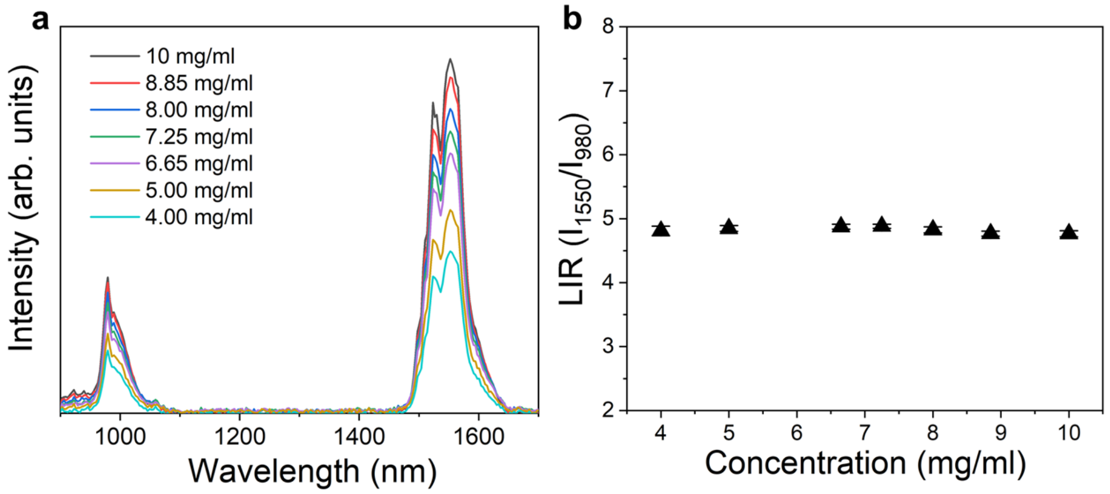

Supplementary Figure 8 | Evaluation of the effect of nanoparticle concentration on the luminescence intensity ratio of LIBRA. (a) Luminescence spectra of LIBRA of varied concentrations in aqueous solutions at room temperature. (b) Luminescence intensity ratio of emissions at 1550 and 980 nm (LIR (I_{1550}/I_{980})) of LIBRA in varied concentrations of aqueous solutions. Data were presented as mean ratio values based

on three measurements (n = 3) of the emission spectra. Error bars were defined as standard deviation.

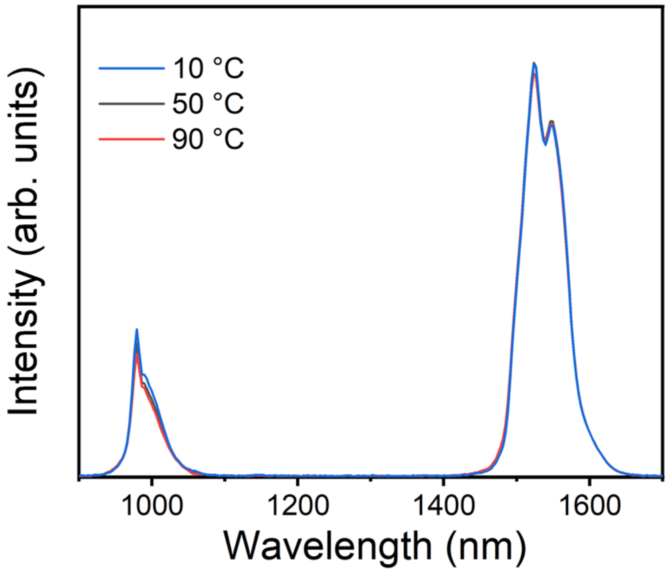

Supplementary Figure 9 | Effect of non-aqueous environment on the temperature response of LIBRA. Near infrared luminescence spectra of LIBRA dispersed in dimethylfuran (DMF) at 10, 50 and 90 °C.

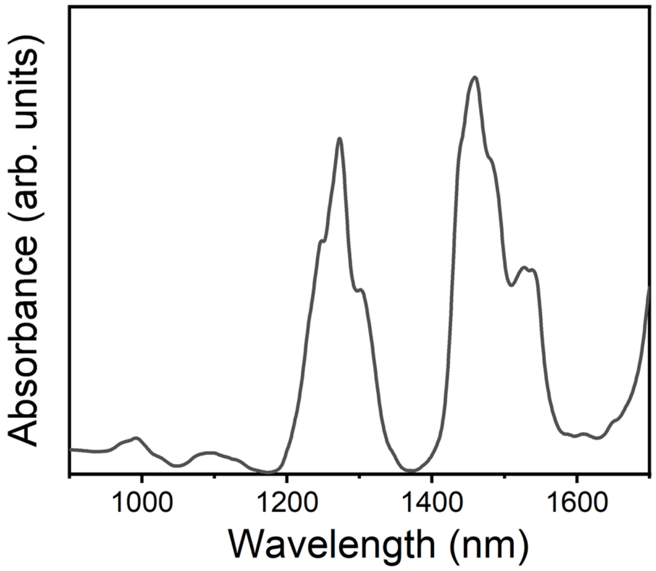

Supplementary Figure 10 | Absorption property of DMF. Absorption spectra of DMF in the near infrared biological window from 900 to 1700 nm.

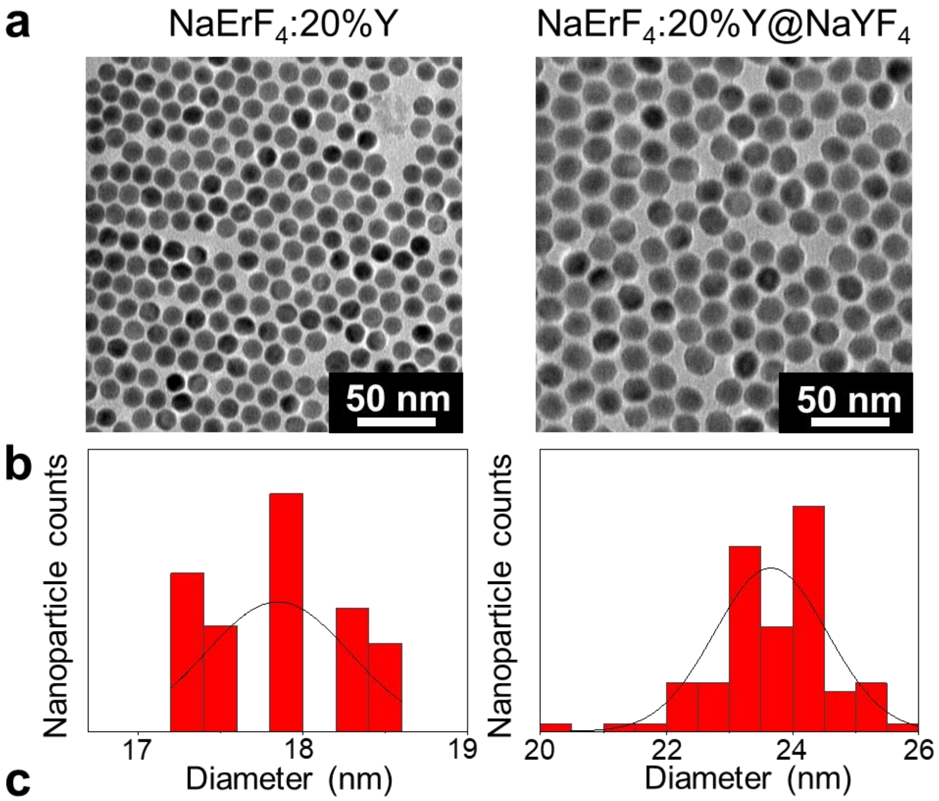

c

	$NaErF_4$:20%Y	$NaErF_4$:20%Y@$NaYF_4$
Diameter (nm)	17.95 ± 0.56	23.66 ± 0.89
Shell thickness (nm)	0	2.86

Supplementary Figure 11 | Morphology characterizations nanoparticles without Yb^{3+} doping. (a) TEM images of $NaErF_4$:20%Y and $NaErF_4$:20%Y@$NaYF_4$. TEM images represent the similar results of experiments repeated independently for three times. (b) Size distribution diagrams of $NaErF_4$:20%Y and $NaErF_4$:20%Y@$NaYF_4$. (c) Statistic diameters and shell thicknesses of $NaErF_4$:20%Y and $NaErF_4$:20%Y@$NaYF_4$.

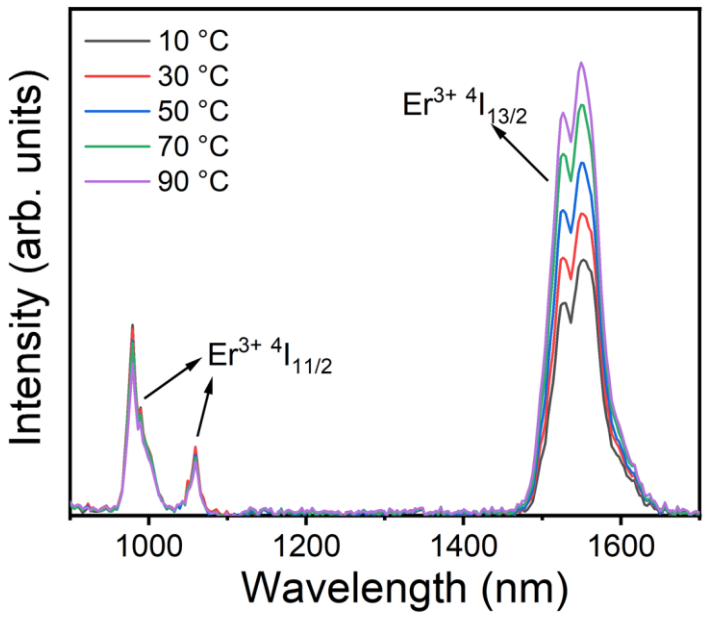

Supplementary Figure 12 | Luminescence property of nanoparticles without Yb^{3+} doping. Emission spectra of $NaErF_4$:20%Y@$NaYF_4$ excited by 808 nm laser in the aqueous solution under varied temperatures.

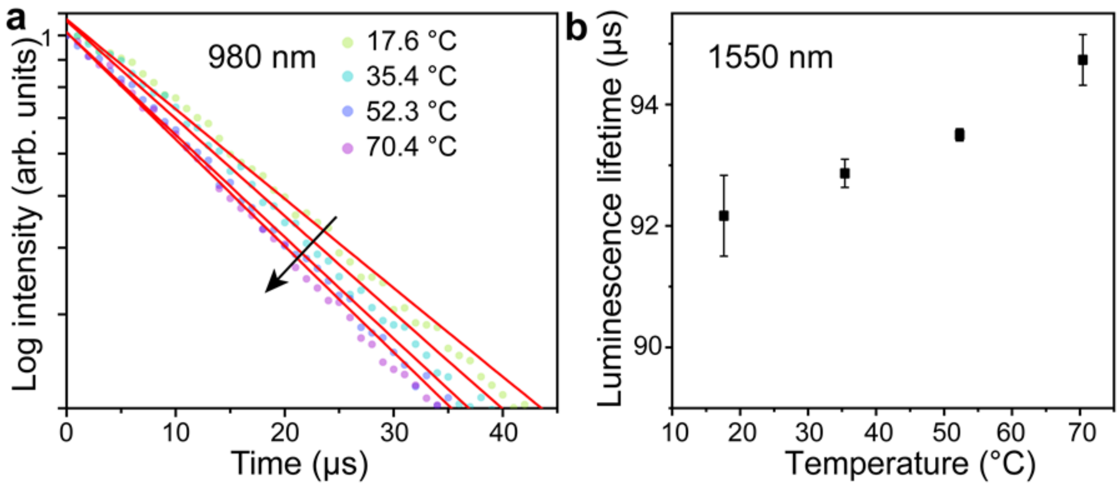

Supplementary Figure 13 | Luminescence lifetime analysis of $NaErF_4$:20%Y@$NaYF_4$. Luminescence lifetime of emissions at (a) 980 nm and (b) 1550 nm in aqueous dispersion at different temperatures. Data were presented as mean lifetime values based on three measurements (n = 3) of the emission spectra. Error bars were defined as standard deviation

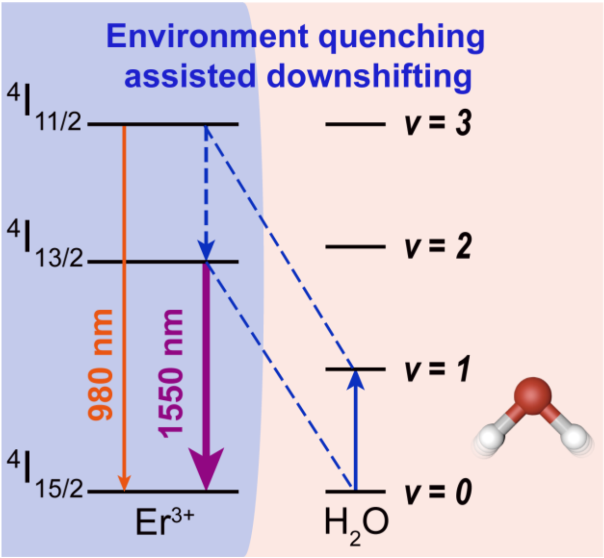

Supplementary Figure 14 | Scheme of thermal response of NaErF4:Y@NaYF4 in aqueous environment. Increased population of $^4I_{13/2}$ state lead to the enhancement of 1550 nm emission and the decreased population of $^4I_{11/2}$ state contributed partially to the suppression of 980 nm emission at high temperature.

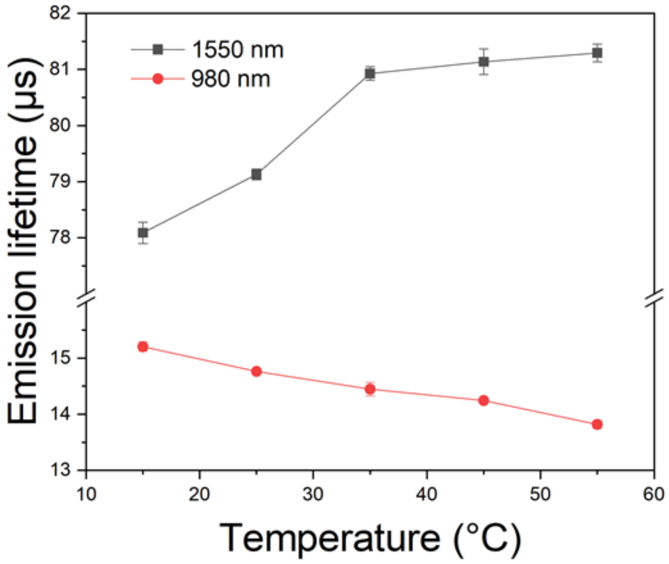

Supplementary Figure 15 | Luminescence lifetime of emissions at 980 nm and 1550 nm of NaErF4:20%Yb@NaYF4:Yb (LIBRA) in aqueous environment under varied temperature. Data were presented as mean emission lifetime values based on three measurements of the luminescence lifetime spectroscopy. Error bars were defined as

standard deviation.

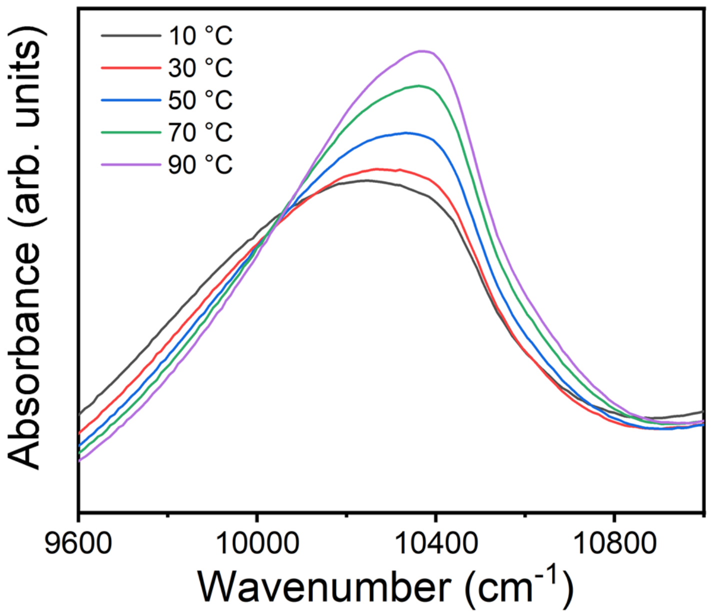

Supplementary Figure 16 | Water absorption spectra from 9600 to 11000 cm^{-1} under varied temperature. The O–H overtone transition $\nu = 0 \rightarrow \nu = 3$ peaked at ~10300 cm^{-1} and increased with temperature.

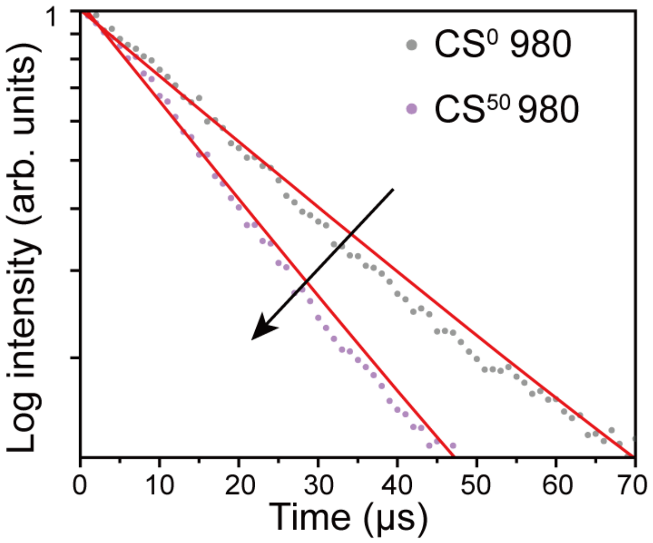

Supplementary Figure 17 | Effect of Yb^{3+} doping in shell layer on the energy migration from nanoparticle to environment. Luminescence decay curve at 980 nm of

nanoparticle with inert shell ($NaErF_4$:Yb@$NaYF_4$, denoted as CS^0) and active shell ($NaErF_4$:Yb@$NaYF_4$:50%Yb, denoted as CS^{50}) in aqueous environment.

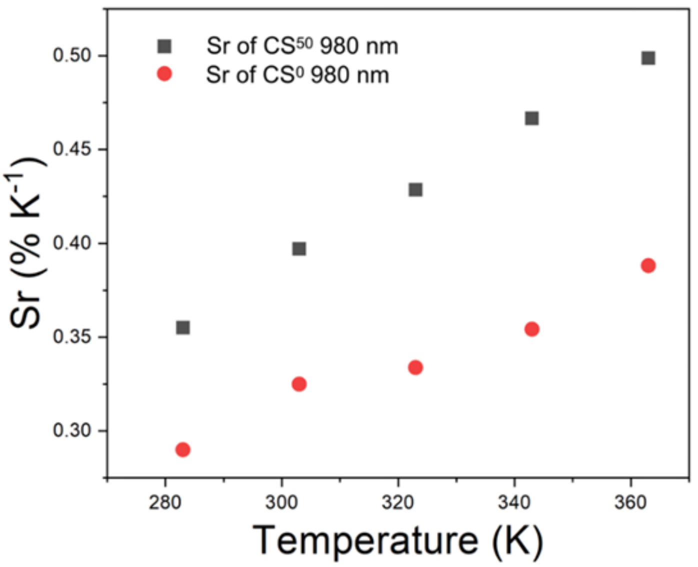

Supplementary Figure 18 | Temperature detection sensitivity of LIBRA. Relative thermal sensitivity (S_r) of 980 nm emission of LIBRA with 0 and 50 % Yb^{3+} in the shell (denoted as CS^0 and CS^{50}, respectively) at different temperature in aqueous dispersion.

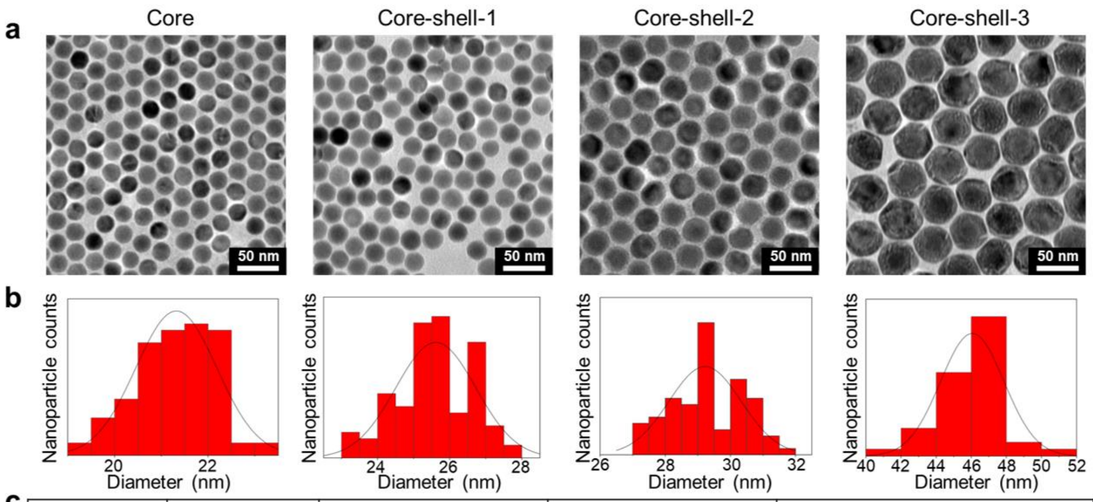

c

	Core $NaErF_4$:20%Yb	Core-shell-1 $NaErF_4$:20%Yb@$NaYF_4$	Core-shell-2 $NaErF_4$:20%Yb@$NaYF_4$	Core-shell-3 $NaErF_4$:20%Yb@$NaYF_4$@$NaLuF_4$
Diameter (nm)	21.32 ± 0.87	25.62 ± 1.11	29.22 ± 1.11	46.06 ± 1.82
Shell thickness (nm)	0	1.6	3.7	12.4

Supplementary Figure 19 | Morphology characterizations of nanoparticles with inert shell layer at different shell thicknesses. (a) TEM images of $NaErF_4$:20%Yb (Core),

and $NaErF_4$:20%Yb@$NaYF_4$ and $NaErF_4$:20%Yb@$NaYF_4$@$NaLuF_4$ (denoted as Core-shell-1, 2 and 3, respectively) with different shell thicknesses. TEM images represent the similar results of experiments repeated independently for three times. (b) Size distribution diagrams of Core and Core-shell-1, 2 and 3. (c) Statistic diameters and shell thicknesses of Core and Core-shell-1, 2 and 3.

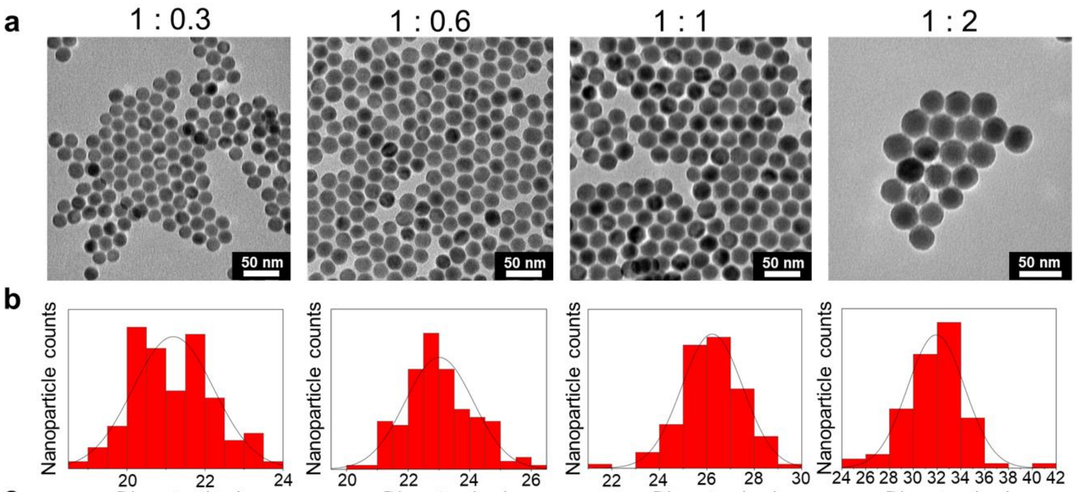

c

CS^{50}	1 : 0.3	1 : 0.6	1 : 1	1 : 2
Diameter (nm)	21.19 ± 1.04	23.01 ± 1.11	26.23 ± 1.29	31.92 ± 2.40
Shell thickness (nm)	0.52	1.45	3.06	5.91

Supplementary Figure 20 | Active shell thickness adjustment of LIBRA. (a) Transmission electron microscopy (TEM) images of LIBRA with various shell thickness of active shell. The adjustment of shell thickness is achieved by adding different molar ratios of lanthanide chloride precursor (50 % YCl_3 and 50 % $YbCl_3$, resulting in LIBRA nanoparticle with the composition of $NaErF_4$:Yb@$NaYF_4$:50%Yb, denoted as CS^{50}) compared to the molar of $NaErF_4$:Yb core nanoparticle for shell layer growth. The molar ratios of the $NaErF_4$:Yb core and the lanthanide chloride precursor for shell growth are 1 : 0.3, 1 : 0.6, 1 : 1 and 1 : 2, respectively. TEM images represent the similar results of experiments repeated independently for three times. (b) Corresponding size distribution diagrams of the LIBRA synthesized with different molar ratios of the $NaErF_4$:Yb core and the lanthanide chloride precursor for shell growth. (c) Statistic particle diameters and shell thicknesses of the nanoparticles in (a).

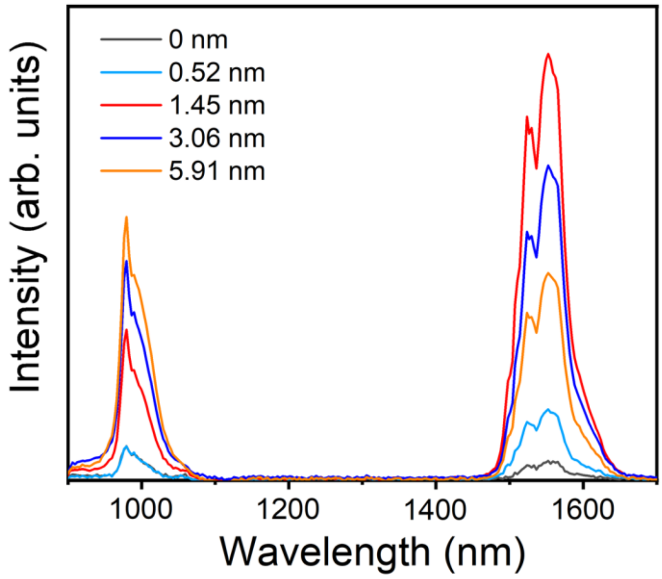

Supplementary Figure 21 | Luminescence property of nanoparticles with active shell at different shell thicknesses. Near infrared luminescence spectra of LIBRA@PEG with the active shell thickness of 0, 0.52, 1.45, 3.06 and 5.91 nm in aqueous environment.

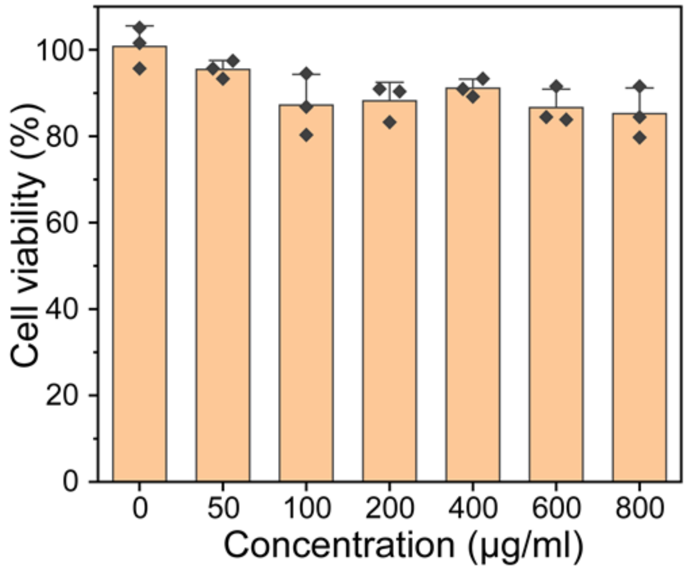

Supplementary Figure 22 | Cytotoxicity of LIBRA. Methyl thiazolyl tetrazolium (MTT) assay of human embryonic kidney (HEK) 293 cells incubated with medium containing different concentration of LIBRA from 0 to 800 μg ml^{-1} to evaluate the cytotoxicity. Data were presented as mean values based on three parallel samples (n = 3). Error bars were defined as standard deviation.

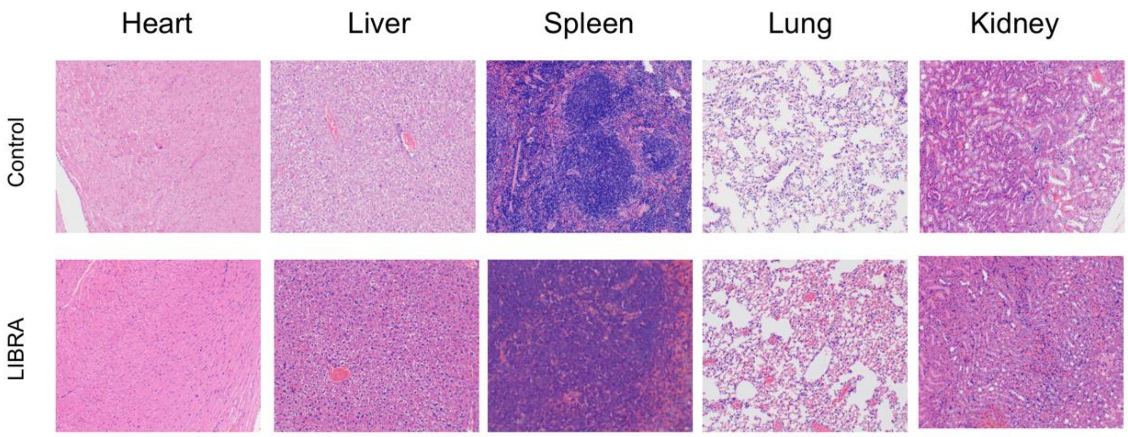

Supplementary Figure 23 | Histology analysis. Haemotoxylin and eosin (H&E) stained histological sections of the main organs (heart, liver, spleen, lung, kidney) from healthy mice without LIBRA injection (Control group) and the mice injected with LIBRA (LIBRA group, 7 days after injection). Histological section images represent the similar results of experiments repeated independently for three times.

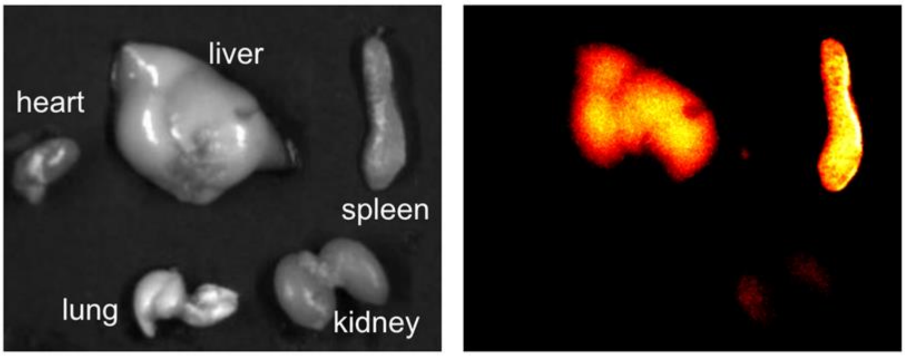

Supplementary Figure 24 | *Ex vivo* near infrared second biological window (NIR-II) imaging. Bright field (left) and NIR-II image (right) of the collected organs from mice 24 h after intravenous injection. The NIR-II image was obtained under excitation of 808 nm laser and 1000 nm long pass filter.

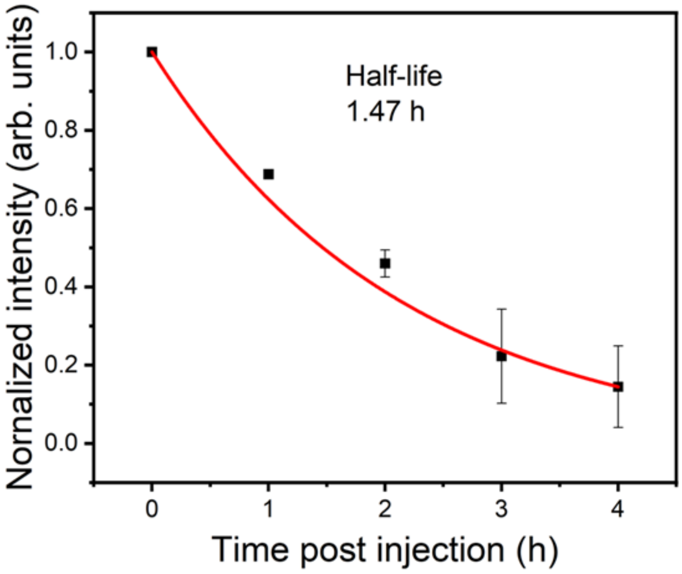

Supplementary Figure 25 | Time-dependent concentration of LIBRA in the blood of Balb/c mice (n = 2) post intravenous injection (based on emission intensity), demonstrating a blood circulation half-life of 1.47 h. Blood samples were collected from orbital venous plexus and mixed with 0.5 mM ethylenediaminetetraacetic acid (EDTA) solution at various time points post injection (up to 4 hours). Data were presented as mean intensity values. Error bars were defined as standard deviation.

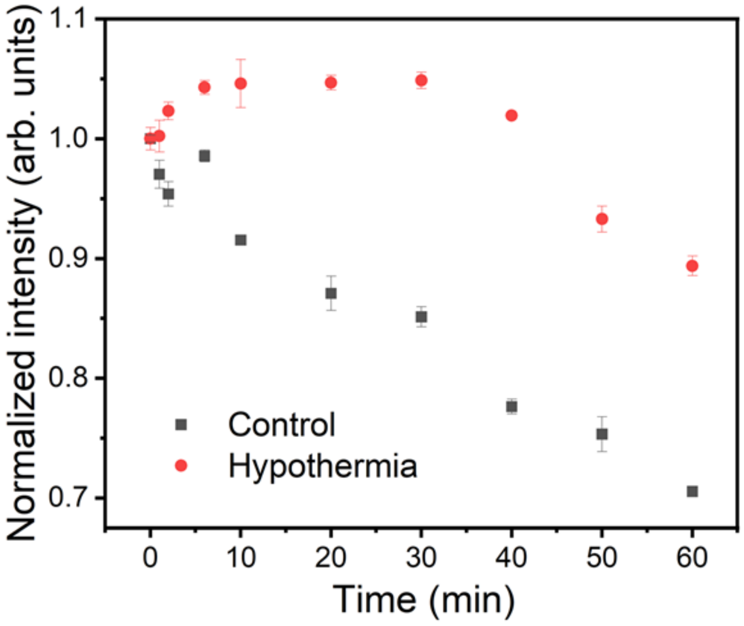

Supplementary Figure 26 | Time-dependent quantitative cerebral vessel luminescence intensity of Control group (black square) and Hypothermia group (red dot) within 60

min. Data were presented as mean intensity values based on the measurement of the luminescence in three mice (n = 3) for each group. Error bars were defined as standard deviation.

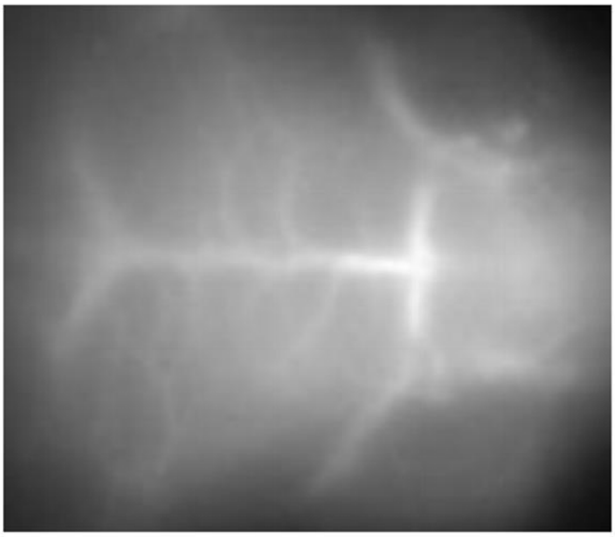

Supplementary Figure 27 | Representative NIR-II imaging of mouse brain under irradiation of 808 nm laser at 120 mW cm^{-2} with 1000 nm long pass filter and an exposure time of 300 ms.

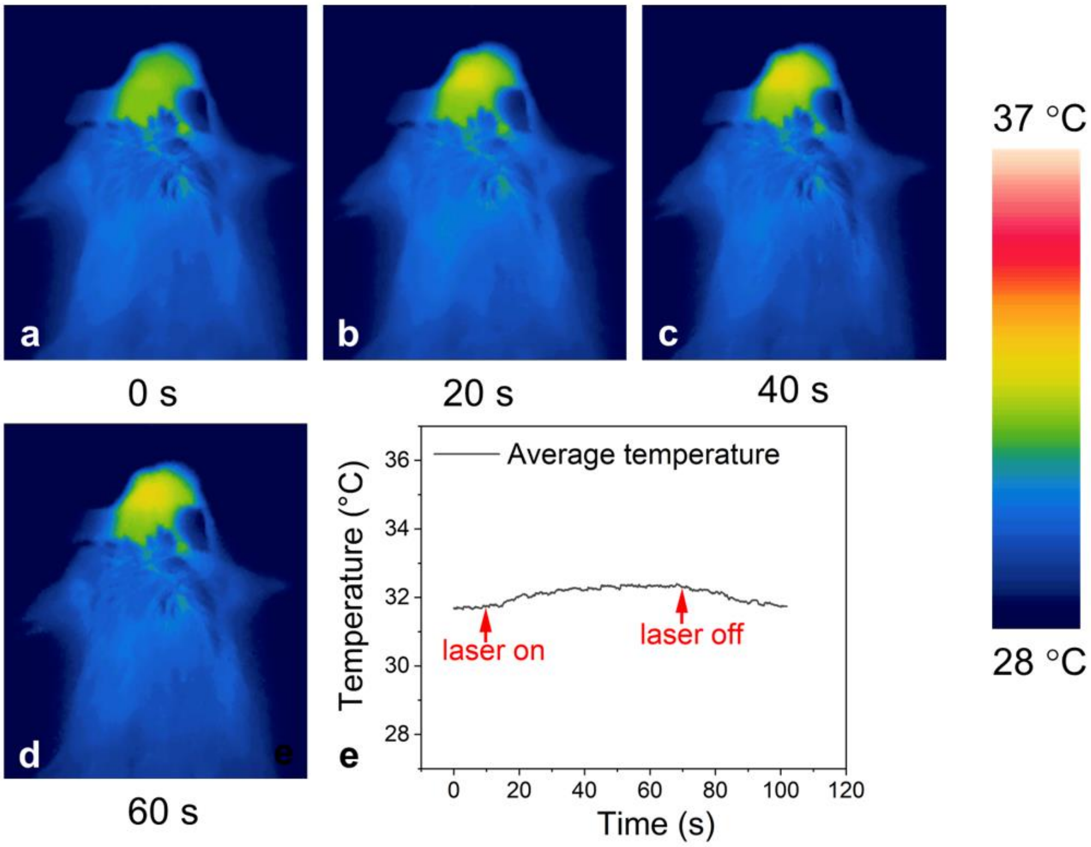

Supplementary Figure 28 | Evaluation of the heating effect of 808 nm laser on mice. (a-d) Time-course thermal images of mouse brain under excitation of 808 nm laser at 120 mW cm^{-2}. (e) Corresponding quantitative temperature variations from thermal images.

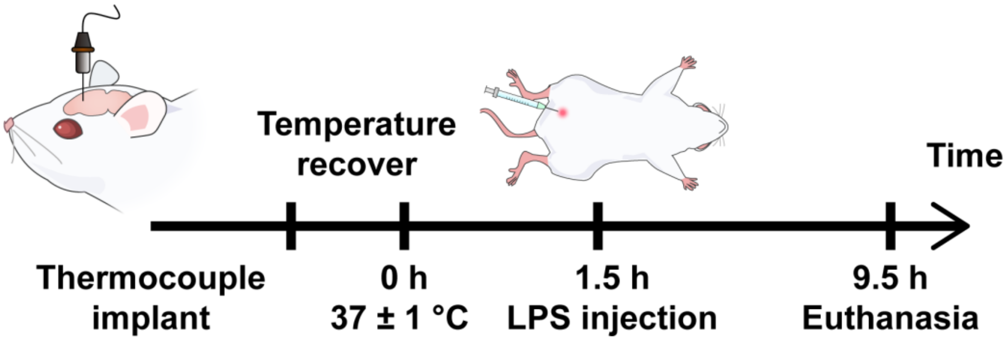

Supplementary Figure 29 | Brain temperature measurement in hypothermia mouse model using thermocouple. Schematic diagram of the timeline and procedures of intracerebral temperature detection through thermocouple before and during lipopolysaccharide (LPS)-induced hypothermia.

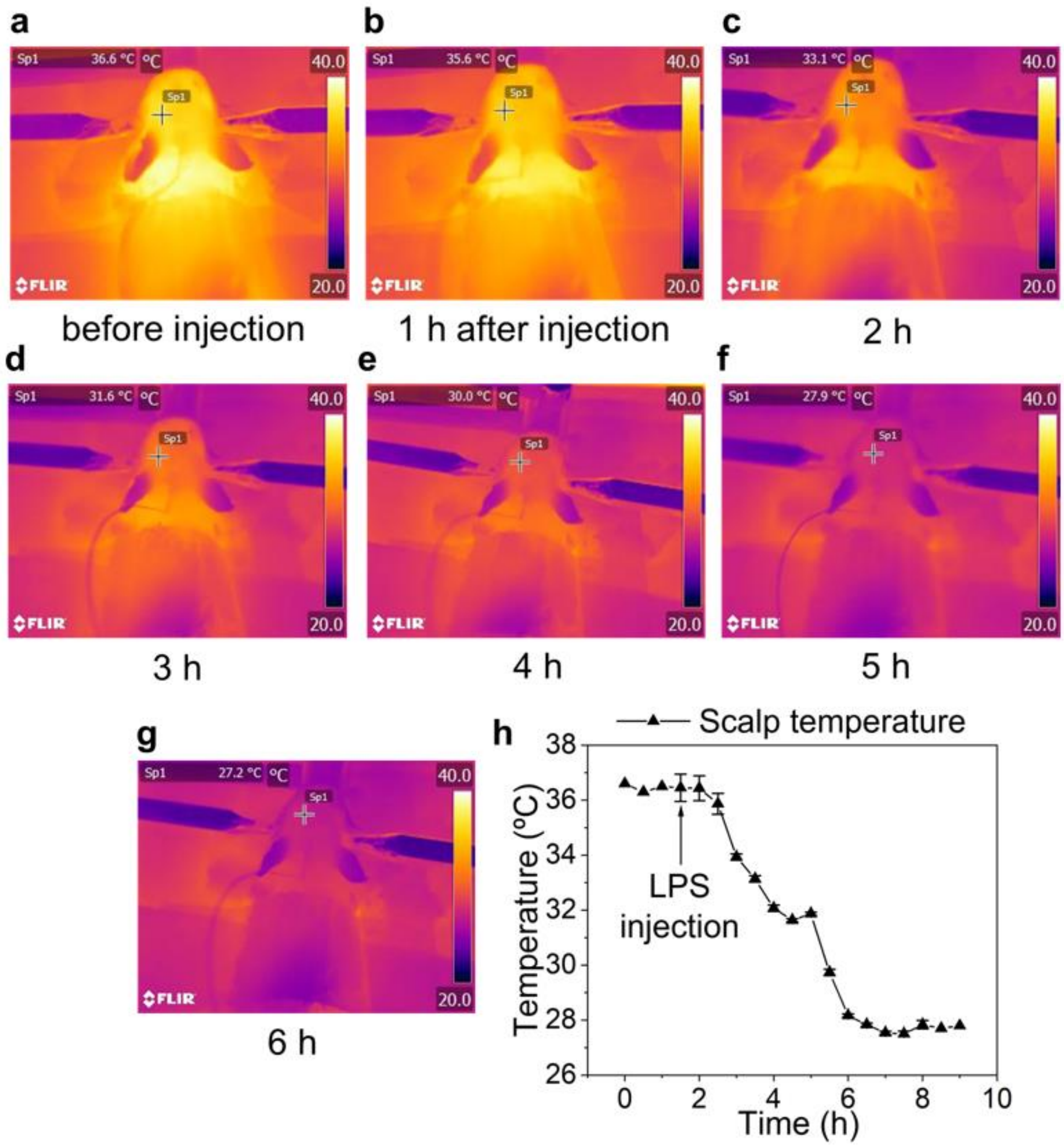

Supplementary Figure 30 | Temperature monitoring of mice using thermal camera with hypothermia model establishment. (a-g) Time-course thermal images of mice before and during LPS-induced hypothermia. (h) Corresponding quantitative temperature variations of scalp from thermal images. Data were presented as mean temperature values based on three mice (n = 3). Error bars were defined as standard deviation.

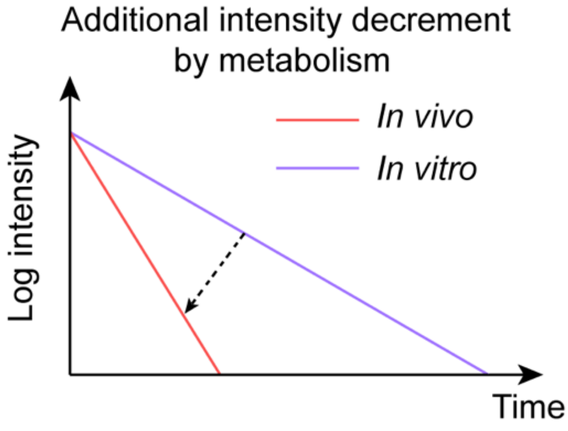

Supplementary Figure 31 | Scheme demonstrating the additional intensity decrement by metabolism. Due to the relatively long collection time of lifetime measurement at current stage, the decrease of nanoparticles luminescence during blood circulation affects the deviation of lifetime detection.

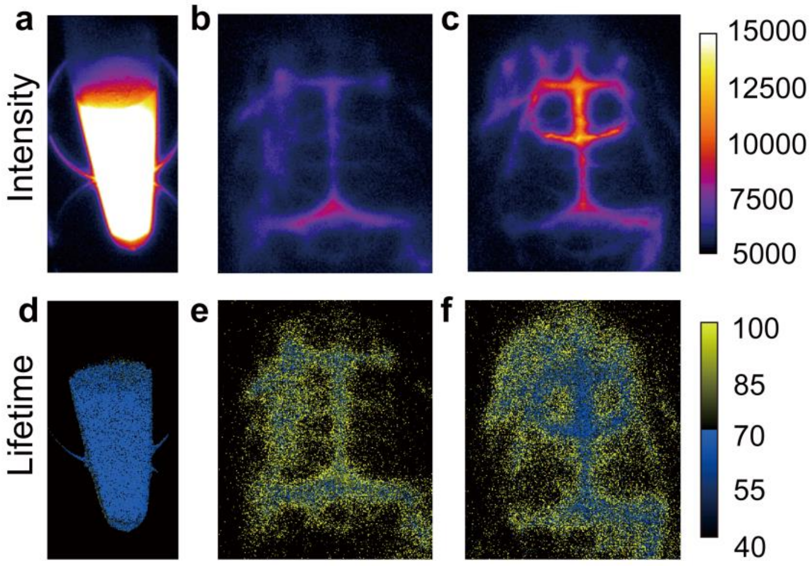

Supplementary Figure 32 | Luminescence intensity imaging beyond 1500 nm of LIBRA in (a) centrifuge tube, (b) mice with cerebral vessel with intact scalp and (c) with sheared scalp. Corresponding luminescence lifetime imaging beyond 1500 nm of LIBRA in (d) centrifuge tube, (e) mice with cerebral vessel with intact scalp and (f) with sheared scalp. The mice were euthanized after LIBRA was injected through caudal vein.

Supplementary Table 1 Parameters of the representative nanothermometer for bio-application reported in scientific papers. λ_{Ex}, λ_{Em}, S_r^{max} correspond to excitation wavelength, working wavelength and the highest relative sensitivity, respectively.

Nanothermometer	$\lambda_{Ex}/\lambda_{Em}$ (nm)	Subject detected	Sensing strategy	S_r^{max} (% K^{-1})	Imaging or not?	Spatial resolution	Reference
Ag_2S	808/1200	Inflamed liver	Lifetime	3	No	-	1
$NaNdF_4$,Yb@CaF_2	785/1000	Leg vessel	Lifetime	~2 (298 K)	Yes	-	2
$NaYF_4$@$NaYF_4$:Yb^{3+}, Nd^{3+}@CaF_2	800/980	Inflamed tissue	Lifetime	1.4 (283 K)	Yes	-	3
$NaYF_4$: Yb^{3+}, Er^{3+}	980/525, 545	Mitochondria in Hela cells	Ratio	3.2 (305 K)	No	-	4
PbS-$NaYbF_4$:Tm@$NaYF_4$:Yb@$NaYF_4$:Nd	865/810	Tumor tissue	Ratio	5.6 (318 K)	Yes	-	5
Ag_2S	808/1200	Hyperthermia liver	Single peak intensity	3.9 (293 K)	Yes		6
Ag_2S	808/1200	Brain tissue	Single peak intensity	3	No	-	7
Er-Yb@Yb-Tm LaF_3	690/1000, 1200	Subcutaneous tissue	Ratio	5	Yes	-	8
PbS/CdS/ZnS	808/1270	Tumor	Single peak intensity	-	No	-	9
$NaErF_4$:Yb@$NaYF_4$:Yb	808/1550, 980	Cerebral vascular	Ratio	1.9	Yes	~200 µm	This work

Supplementary Table 2 Temperature reading methods used for biomedical application.

Temperature imaging techniques	Biological model	Stimuli	Surface or interior	Real time or not?	Reference
Infrared thermal imaging	Mouse tumor	Photothermal therapy	Surface	Yes	10
Magnetic resonance spectroscopy	Human brain	-	Interior	No	11
Magnetic resonance spectroscopy	Human brain	Acute ischemic stroke	Interior	No	12
Magnetic resonance spectroscopy	Human brain	Ice slurry ingestion	Interior	No	13
Photoacoustic imaging	Human prostate	Cryotherapy	Interior	Yes	14
Photoacoustic imaging	Ex-vivo bovine tissue	High-intensity focused ultrasound therapy	Interior	Yes	15
Nanothermometer	Mouse limb vessel	Heating pad	Interior	Yes	2
Nanothermometer	Mouse hyperthermia liver	Laser heating	Interior	Yes	16
Nanothermometer	Mouse Subcutaneous tissue	Laser heating	Interior	Yes	8
Optical fiber	Mouse brain	Freely behaving mouse	Interior	Yes	17,18
Nanothermometer	Mouse brain	Hypothermia induced by LPS	Interior	Yes	This work

Supplementary Notes 1

Establishment of light propagation model for calibrating the relationship between luminescence intensity ratio and temperature

1. Diffuse light propagation in the medium with scattering and absorption

Diffuse light propagation in the medium with scattering and absorption can be

predicted by using a photon diffusion equation[19, 20] given as,

$$\frac{\partial \Phi(\vec{r},t)}{c\partial t} + \mu_a \Phi(\vec{r},t) - \nabla \cdot [D\nabla\Phi(\vec{r},t)] = S(\vec{r},t) \quad (1)$$

where $\Phi(\vec{r},t)$ denotes fluence rate (or intensity) of light at location $\vec{r}$ and time point t. The medium is characterized by its absorption coefficient (μ_a) and reduced scattering coefficient (μ_s'). On the right side of the equation, $S(\vec{r},t)$ is the source term. Furthermore, D is referred to as the diffusion coefficient, which can be obtained from,

$$D = \frac{1}{3(\mu_a + \mu_s')} \quad (2)$$

For a time-independent point source, $S(\vec{r},t) = \delta(\vec{r})$, the solution to Eq. (1) is typically known as the Green's function,

$$\Phi(\vec{r}) = \frac{1}{4\pi D r} e^{-\mu_{eff} \cdot \vec{r}} \quad (3)$$

where μ_{eff} denotes the effective attenuation coefficient that is expressed as,

$$\mu_{eff} = \sqrt{\frac{\mu_a}{D}} = \sqrt{3\mu_a(\mu_a + \mu_s')} \quad (4)$$

2. Modeling and determination of the relationship between luminescence intensity ratio (LIR) and temperature

To give the accurate relationship of the luminescence intensity ratio (LIR) and temperature in the biological tissue, it is necessary to establish the model that describes the problem. In the model, as illustrated in Supplementary Fig. 33, the nanothermometer (LIBRA) as luminescence source is embedded in the biological tissue (medium). The incident excitation propagates in the medium and irradiates the luminescence source in the medium at depth d. The luminescence propagates in the medium and is measured by the detector on the top surface of the medium right above the luminescence source. The solution process is divided into three stages: 1) solving for the fluence intensity of incident excitation in the medium, 2) solving for the luminescence intensity of the luminescence source propagating in the medium, and 3) establishing the relationship of LIR and temperature in the medium.

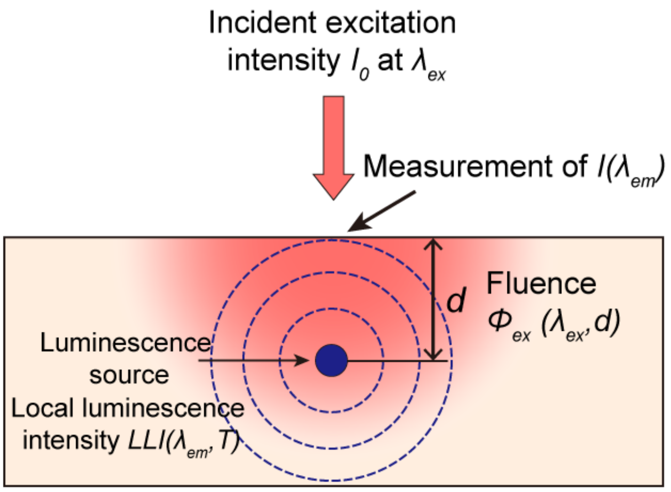

Supplementary Figure 33 | Schematic of excitation and luminescence propagations in a medium with scattering and absorption. Incident excitation with a fluence intensity, $\Phi_{ex}(\lambda_{ex}, d)$, excites the luminescence source (green) embedded at a depth of d inside the medium to generate the local luminescence intensity, $LLI(\lambda_{em}, T)$. The luminescence propagates in the medium and is measured on the top surface of the medium to be $I(\lambda_{em})$.

2.1 Fluence intensity of incident excitation

For incident excitation with initial power I_0 at wavelength λ_{ex}, the Green's function solution of diffusion equation (Eq. 1) can be used to determine the excitation light field $\Phi_{ex}(\lambda_{ex}, d)$ at depth d,

$$\Phi_{ex}(\lambda_{ex}, d) = I_0 \cdot \frac{1}{4\pi D(\lambda_{ex}) \cdot d} e^{-\mu_{eff}(\lambda_{ex})d} \tag{5}$$

2.2 Local luminescence intensity (LLI)

Afterwards, local luminescence intensity (LLI), which is the original intensity emitted by the luminescence source, is calculated. This is the result of excitation field, the absorption of luminescence source and its quantum yield. At a given temperature (T), LLI of the emission at wavelength λ_{em} is as follows:

$$LLI(\lambda_{em}, T) = \Phi_{ex}(\lambda_{ex}, d) \cdot \mu_{a,l}(\lambda_{ex}) \cdot \sigma(\lambda_{em}, T) \tag{6}$$

where $\Phi_{ex}(\lambda_{ex}, d)$ is the excitation field, $\mu_{a,l}(\lambda_{ex})$ is the absorption of the luminescence source, and $\sigma(\lambda_{em}, T)$ is its corresponding quantum yield.

For the ratiometric thermometry in this work, the temperature is correlated with the ratio of LLI of the emissions at two different wavelengths (λ_{em1} and λ_{em2}), which

is also equal to the ratio of quantum yields (σ) of the two emissions. The linear relationship between the ratio of LLI and temperature (T) can be written as the following form,

$$T = \alpha \cdot \frac{\sigma(\lambda_{em2},T)}{\sigma(\lambda_{em1},T)} + \beta \quad (7)$$

where α and β are constant, λ_{em1} and λ_{em2} in this work are the emissions at 980 and 1550 nm, respectively.

2.3 Luminescence intensity after light propagation in the medium

The luminescence intensity of the luminescence source after light propagation in the medium, $I(\lambda_{em})$, is acquired in the experiment. Again, the Green function is used to describe $I(\lambda_{em})$ of emission at wavelength λ_{em}, which refers to $LLI(\lambda_{em},T)$:

$$I(\lambda_{em}) = LLI(\lambda_{em},T) \cdot \frac{1}{4\pi D(\lambda_{em})\cdot d} e^{-\mu_{eff}(\lambda_{em})d} \quad (8)$$

By substituting $LLI(\lambda_{em},T)$ in Eq. 8 with Eq. 5 and 6, $I(\lambda_{em})$ can be expressed as:

$$I(\lambda_{em}) = \frac{I_0}{16\pi^2 d^2 D(\lambda_{ex})D(\lambda_{em})} e^{-\left(\mu_{eff}(\lambda_{ex})+\mu_{eff}(\lambda_{em})\right)\cdot d} \cdot \mu_{a,l}(\lambda_{ex}) \cdot \sigma(\lambda_{em},T) \quad (9)$$

2.4 LIR after light propagation in the medium

After light propagation in the medium, the luminescence intensities of the emissions at two wavelengths, $I(\lambda_{em1})$ and $I(\lambda_{em2})$, can be described by Eq. 9 and are measured in the experiment. The ratio of $I(\lambda_{em1})$ and $I(\lambda_{em2})$ is denoted as R_I that is,

$$R_I = \frac{I(\lambda_{em2})}{I(\lambda_{em1})}$$
$$= \frac{\frac{I_0}{16\pi^2 d^2 D(\lambda_{ex})D(\lambda_{em2})} e^{-\left(\mu_{eff}(\lambda_{ex})+\mu_{eff}(\lambda_{em2})\right)\cdot d} \cdot \mu_{a,f}(\lambda_{ex})\cdot\sigma(\lambda_{em2},T)}{\frac{I_0}{16\pi^2 d^2 D(\lambda_{ex})D(\lambda_{em1})} e^{-\left(\mu_{eff}(\lambda_{ex})+\mu_{eff}(\lambda_{em1})\right)\cdot d} \cdot \mu_{a,f}(\lambda_{ex})\cdot\sigma(\lambda_{em1},T)}$$
$$= \frac{D(\lambda_{em1})}{D(\lambda_{em2})} e^{(\mu_{eff}(\lambda_{em1})-\mu_{eff}(\lambda_{em2}))\cdot d} \cdot \frac{\sigma(\lambda_{em2},T)}{\sigma(\lambda_{em1},T)} \quad (10)$$

Based on Eq. 7, the ratio of quantum yields of λ_{em2} and λ_{em1} can be expressed as,

$$\frac{\sigma(\lambda_{em2},T)}{\sigma(\lambda_{em1},T)} = \frac{T-\beta}{\alpha} \quad (11)$$

A correction term, γ, is further defined that is given as:

$$\gamma = \frac{D(\lambda_{em1})}{D(\lambda_{em2})} e^{(\mu_{eff}(\lambda_{em1}) - \mu_{eff}(\lambda_{em2}))\cdot d} \quad (12)$$

By substituting the corresponding items with Eq. 11 and 12, Eq. 10 can be expressed as,

$$R_I = \frac{T - \beta}{\alpha} \cdot \gamma$$

or:

$$T = \frac{\alpha}{\gamma} R_I + \beta \quad (13)$$

The term γ is related to the absorption and scattering of the medium at certain depth, which is used to correct the correlation of temperature (T) and the ratio of the luminescence intensities ($I(\lambda_{em})$) detected in the experiment that are derived from the local luminescence intensity of LIBRA nanothermometers and propagate in the biological tissue. Thus, the accurate relationship between LIR and temperature can be established as Eq. 13.

3. Validation of modeling

3.1 Phantom preparation and characterizations

To validate our model, a series of phantoms (Phantom A, B, C, D) with determined and stable thicknesses and optical properties (absorption coefficient (μ_a) and reduced scattering coefficient (μ_s')) were prepared and used for measuring the relationship of temperature and the LIR of LIBRA nanothermometer (Supplementary Fig. 34). The phantoms consist of polydimethylsiloxane (PDMS) elastomer (SYLGARDTM 184, DOW Corporation, America) as matrix and the μ_a and μ_s' of phantoms are adjusted by integrating different levels of carbon black powder (Product No. 7440-44-0, Collins, China) and titanium dioxide (Product No. 13463-67-7, Collins, China), respectively. The optical properties at 980 nm and 1550 nm of the phantoms are calibrated using inverse adding-doubling (IAD) method[21]. The detailed information about the phantoms is given in Supplementary Table 3.

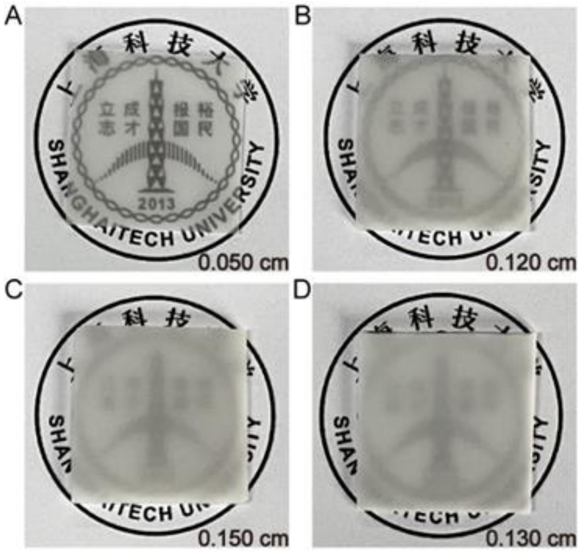

Supplementary Figure 34 | Images of Phantom A, B, C and D with different optical properties. Optical parameters include absorption coefficient (μ_a) and reduced scattering coefficient (μ_s')) and thicknesses.

Supplementary Table 3 Thicknesses and optical properties (absorption coefficient (μ_a) and reduced scattering coefficient (μ_s')) of phantoms.

Phantom	A	B	C	D
Thickness (cm)	0.050	0.120	0.150	0.130
μ_a (cm^{-1}), 980 nm	1.075	0.615	0.496	0.479
μ_s' (cm^{-1}), 980 nm	6.133	5.662	5.584	7.956
μ_a (cm^{-1}), 1550 nm	2.729	2.430	2.372	2.239
μ_s' (cm^{-1}), 1550 nm	2.628	2.486	2.371	4.321

3.2 Measurement of temperature dependent luminescence intensity ratio with different phantoms based on NIR imaging

To validate the feasibility of the proposed function (Eq. 13) for describing the relationship of luminescence intensity ratio (LIR) and temperature with the condition of absorption and scattering, the experiments for measuring the LIR of LIBRA nanothermometer were performed at a series of temperature with the use of Phantom A, B, C, D. The experimental setup was depicted in Supplementary Fig. 35. A

continuous-wave (CW) 808 nm laser coupled with a collimator (LE-LS-808-XXFC, Leoptics Inc., China) and a diffuser (ED1-C20, Thorlabs, New Jersey, USA) was employed as excitation source. LIBRA dispersed in aqueous solution was added into a capillary tube and the phantom was covered on the top. A high-precision heater (MDB100C, Joanlab Inc., China) was used for controlling temperature of LIBRA dispersion within a range of 26-46 °C and a thermocouple was inserted in the aqueous dispersion for real-time temperature recording. NIR-II/III imaging system (Monet IGS-1000P, Suzhou NIR-Optics Co., Ltd., China) was used to record the images collected by 900 nm and 1400 nm long pass filters to calculate the LIR of the emission bands at 980 nm and 1550 nm. During data acquisition, the temperature of LIBRA dispersion was controlled continuously and the NIR-II/III images were acquired at a frame rate of 1 fps and the exposure time was set to 200 ms. The thermocouple recorded the temperature change simultaneously with a sampling interval of 1 s.

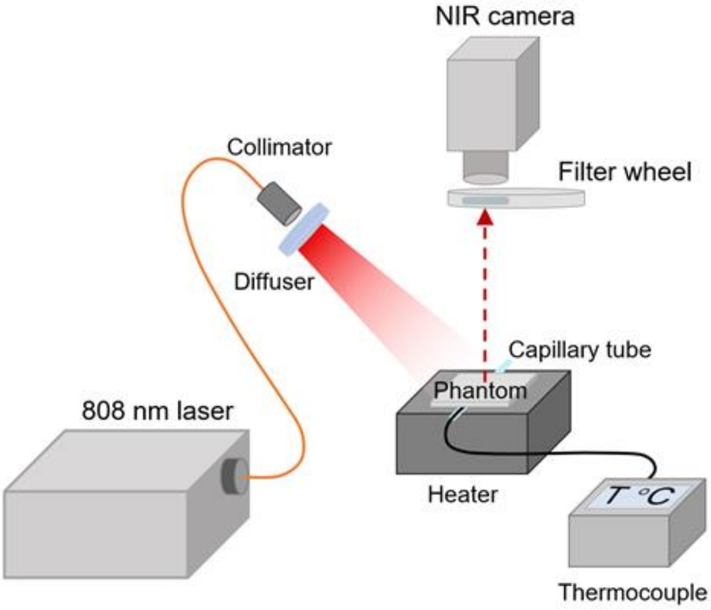

Supplementary Figure 35 | Light propagation validation method. Schematic diagram of the experimental setup for measurement of temperature dependent luminescence intensity ratio with different phantoms based on NIR-II/III imaging.

3.3 Feasibility evaluation and establishment of the calibrated equation for luminescence intensity ratio and temperature correlation *in vivo*

The LIR of the emissions at 1550 and 980 nm (I_{1550}/I_{980}, namely R_I) acquired by

the NIR-II/III imaging results and the temperature detected simultaneously were integrated together showing a linear correlation. On the other hand, the relationship of LIR and temperature was computed by using Eq. 13. The parameters of α and β in Eq. 13 were 15.5 and -66.5, respectively, that were fixed in each phantom and solved based on the experimental measurements. The correction term γ for Phantom A to D were calculated according to the thicknesses and optical properties of different phantoms that were 0.71, 0.59, 0.54 and 0.53, respectively. The fitting curves describing the LIR and temperature relationship for different phantoms are presented as: $T = 21.8\ R_I - 66.5$ (Phantom A); $T = 26.3\ R_I - 66.5$ (Phantom B); $T = 28.7\ R_I - 66.5$ (Phantom C); $T = 29.2\ R_I - 66.5$ (Phantom D). As shown in Supplementary Fig. 36, the fitting curves matched well with the experimental results (correlation coefficient >0.990), which indicated that the established model can accurately describe the effects of medium with absorption and scattering on the luminescence signals, and the proposed equation to calibrate the LIR and temperature relationship is feasible. As the light extinction parameters of the biological tissues including skin, scalp, blood, etc. were determined in reported works[22, 23], the calibrated equation can be calculated as $T = 22.9\ R_I - 66.5$ that is for the temperature detection of mice cerebral vessels *in vivo*.

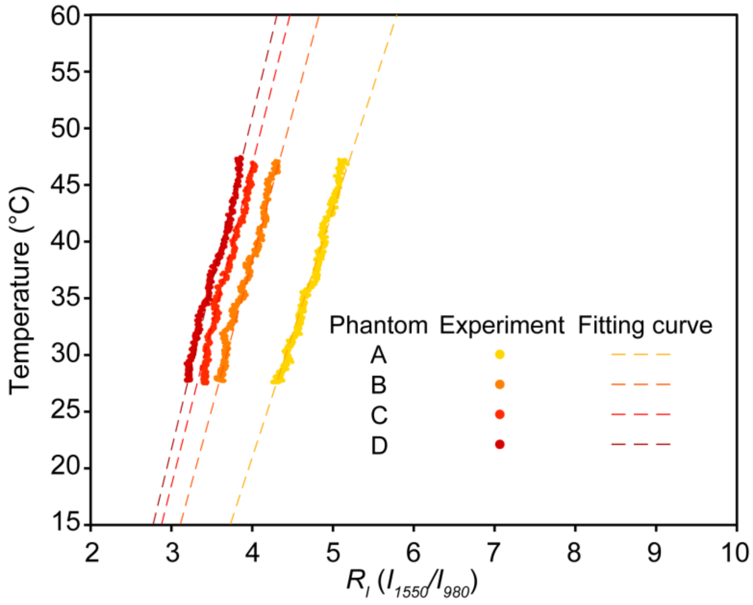

Supplementary Figure 36 | Light propagation measurement. Experimental results and fitting curves of the relationship of luminescence intensity ratio R_I (I_{1550}/I_{980}) and temperature in different phantoms.

Reference

1 Shen, Y. *et al.* Reliable and Remote Monitoring of Absolute Temperature during Liver Inflammation via Luminescence-Lifetime-Based Nanothermometry. *Adv. Mater.* **34**, e2107764 (2022).

2 Kong, M. *et al.* Luminescence interference-free lifetime nanothermometry pinpoints in vivo temperature. *Sci. China Chem.* **64**, 974-984 (2021).

3 Tan, M. *et al.* Accurate In Vivo Nanothermometry through NIR-II Lanthanide Luminescence Lifetime. *Small* **16**, 2004118 (2020).

4 Di, X. *et al.* Quantitatively Monitoring In Situ Mitochondrial Thermal Dynamics by Upconversion Nanoparticles. *Nano Lett.* **21**, 1651-1658 (2021).

5 Qiu, X. *et al.* Ratiometric upconversion nanothermometry with dual emission at the same wavelength decoded via a time-resolved technique. *Nat. Commun.* **11**, 4 (2020).

6 Lifante, J. *et al.* Reaching Deeper: Absolute In Vivo Thermal Reading of Liver by Combining Superbright Ag_2S Nanothermometers and In Silico Simulations. *Adv. Sci.* **8**, 2003838 (2021).

7 del Rosal, B. *et al.* In Vivo Contactless Brain Nanothermometry. *Adv. Func. Mater.* **28**, 1806088 (2018).

8 Ximendes, E. C. *et al.* In Vivo Subcutaneous Thermal Video Recording by Supersensitive Infrared Nanothermometers. *Adv. Func. Mater.* **27**, 1702249 (2017).

9 del Rosal, B. *et al.* Infrared-Emitting QDs for Thermal Therapy with Real-Time Subcutaneous Temperature Feedback. *Adv. Func. Mater.* **26**, 6060-6068 (2016).

10 Robinson, J. T. *et al.* High performance in vivo near-IR (>1 μm) imaging and photothermal cancer therapy with carbon nanotubes. *Nano Res.* **3**, 779-793 (2010).

11 Sung, D. *et al.* Personalized predictions and non-invasive imaging of human brain temperature. *Commun. Phys.* **4**, 68 (2021).

12 Karaszewski, B. *et al.* Measurement of brain temperature with magnetic

resonance spectroscopy in acute ischemic stroke. *Ann. Neurol.* **60**, 438-446 (2006).

13 Onitsuka, S. *et al.* Ice slurry ingestion reduces human brain temperature measured using non-invasive magnetic resonance spectroscopy. *Sci. Rep.* **8**, 2757 (2018).

14 Petrova, E. V., Brecht, H. P., Motamedi, M., Oraevsky, A. A. & Ermilov, S. A. In vivo optoacoustic temperature imaging for image-guided cryotherapy of prostate cancer. *Phys. Med. Biol.* **63**, 064002 (2018).

15 Xun, W., Sanders, J. L., Stephens, D. N. & Oralkan, O. Photoacoustic-imaging-based temperature monitoring for high-intensity focused ultrasound therapy. *Annu. Int. Conf. IEEE Eng. Med. Biol. Soc.* **2016**, 3235-3238 (2016).

16 Lifante, J. *et al.* Reaching Deeper: Absolute In Vivo Thermal Reading of Liver by Combining Superbright Ag_2S Nanothermometers and In Silico Simulations. *Adv. Sci.* **8**, 2003838 (2021).

17 Fedotov, I. V. *et al.* All-Optical Brain Thermometry in Freely Moving Animals. *ACS Photonics* **7**, 3353-3360 (2020).

18 Ding, H. *et al.* An Optoelectronic thermometer based on microscale infrared-to-visible conversion devices. *Light Sci. Appl.* **11**, 130 (2022).

19 Arridge, S. R., Optical tomography in medical imaging. *Inverse Probl.* **15**, R41 (1999).

20 Marcu, L., French, P. M., & Elson, D. S. (Eds.), *Fluorescence Lifetime Spectroscopy and Imaging: Principles and Applications in Biomedical Diagnostics*. CRC press, Boca Raton (2014).

21 Pickering, J. W. *et al.* Double-integrating-sphere system for measuring the optical properties of tissue. *Appl. Opt.* **32**, 399-410 (1993).

22. Hong G, *et al.* Through-skull fluorescence imaging of the brain in a new near-infrared window. *Nat. Photonics* **8**, 723-730 (2014).

23. Friebel M, Helfmann J, Netz U, Meinke M. Influence of oxygen saturation on the optical scattering properties of human red blood cells in the spectral range 250 to 2000 nm. *J. Biomed. Opt.* **14**, 034001 (2009).
