# Supplementary material for: Lanthanide luminescence nanothermometer with working wavelength beyond 1500 nm for cerebrovascular temperature imaging in vivo
Source: Nat Commun. 2024 Mar 15;15:2341. doi: 10.1038/s41467-024-46727-5 (PMC10943110; doi:10.1038/s41467-024-46727-5)
Supplement: Supplementary file 3 — Reporting Summary [file 41467_2024_46727_MOESM3_ESM.pdf]

nature portfolio

Corresponding author(s): Xingjun Zhu

Last updated by author(s): 2024-02-07

Reporting Summary

Nature Portfolio wishes to improve the reproducibility of the work that we publish. This form provides structure for consistency and transparency in reporting. For further information on Nature Portfolio policies, see our Editorial Policies and the Editorial Policy Checklist.

Statistics

For all statistical analyses, confirm that the following items are present in the figure legend, table legend, main text, or Methods section.

n/a	Confirmed	
☐	☐	The exact sample size (n) for each experimental group/condition, given as a discrete number and unit of measurement
☐	☒	A statement on whether measurements were taken from distinct samples or whether the same sample was measured repeatedly
☒	☐	The statistical test(s) used AND whether they are one- or two-sided<br>*Only common tests should be described solely by name; describe more complex techniques in the Methods section.*
☒	☐	A description of all covariates tested
☒	☐	A description of any assumptions or corrections, such as tests of normality and adjustment for multiple comparisons
☐	☒	A full description of the statistical parameters including central tendency (e.g. means) or other basic estimates (e.g. regression coefficient) AND variation (e.g. standard deviation) or associated estimates of uncertainty (e.g. confidence intervals)
☒	☐	For null hypothesis testing, the test statistic (e.g. F, t, r) with confidence intervals, effect sizes, degrees of freedom and P value noted<br>*Give P values as exact values whenever suitable.*
☒	☐	For Bayesian analysis, information on the choice of priors and Markov chain Monte Carlo settings
☒	☐	For hierarchical and complex designs, identification of the appropriate level for tests and full reporting of outcomes
☒	☐	Estimates of effect sizes (e.g. Cohen's d, Pearson's r), indicating how they were calculated

Our web collection on statistics for biologists contains articles on many of the points above.

Software and code

Policy information about availability of computer code

Data collection	Origin (Originlab Corp.), ImageJ, Gatan Microscopy Suite Software are used to collect the spectroscopy and graphical data in this work.
Data analysis	Origin (OriginLab Corp.), ImageJ are used to analyze the spectroscopy and graphical data in this work.

For manuscripts utilizing custom algorithms or software that are central to the research but not yet described in published literature, software must be made available to editors and reviewers. We strongly encourage code deposition in a community repository (e.g. GitHub). See the Nature Portfolio guidelines for submitting code & software for further information.

Data

Policy information about availability of data

All manuscripts must include a data availability statement. This statement should provide the following information, where applicable:

- Accession codes, unique identifiers, or web links for publicly available datasets
- A description of any restrictions on data availability
- For clinical datasets or third party data, please ensure that the statement adheres to our policy

Al the data generated and analyzed from this study are presented in the article and its Supplementary information files and Source Data file. The data are also available from the corresponding author upon request.

Research involving human participants, their data, or biological material

Policy information about studies with human participants or human data. See also policy information about sex, gender (identity/presentation), and sexual orientation and race, ethnicity and racism.

Reporting on sex and gender	Not applicable.
Reporting on race, ethnicity, or other socially relevant groupings	Not applicable.
Population characteristics	Not applicable.
Recruitment	Not applicable.
Ethics oversight	Not applicable.

Note that full information on the approval of the study protocol must also be provided in the manuscript.

Field-specific reporting

Please select the one below that is the best fit for your research. If you are not sure, read the appropriate sections before making your selection.

☒ Life sciences ☐ Behavioural & social sciences ☐ Ecological, evolutionary & environmental sciences

For a reference copy of the document with all sections, see nature.com/documents/nr-reporting-summary-flat.pdf

Life sciences study design

All studies must disclose on these points even when the disclosure is negative.

Sample size	Sample size was determined by the difference between two groups including the control group and the hypothermia model group. The temperature of the hypothermia group has an average of 8.5 °C decrease compared to the temperature of control group with a standard deviation of +1 °C and thus the sample size of 3 is enough to discriminate the hypothermia symptom.
Data exclusions	No data is excluded from the analyses.
Replication	All attempts at replication were successful.
Randomization	7 weeks old female Balb/c mice were randomly allocated into the control group without the establishment of hypothermia model and experiment group with the establishment of hypothermia model for the following experiment.
Blinding	The investigators were blinded to group allocation during data collection and analysis.

Reporting for specific materials, systems and methods

We require information from authors about some types of materials, experimental systems and methods used in many studies. Here, indicate whether each material, system or method listed is relevant to your study. If you are not sure if a list item applies to your research, read the appropriate section before selecting a response.

Materials & experimental systems

n/a	Involved in the study	
☒	☐	Antibodies
☐	☒	Eukaryotic cell lines
☒	☐	Palaeontology and archaeology
☐	☒	Animals and other organisms
☒	☐	Clinical data
☒	☐	Dual use research of concern
☒	☐	Plants

Methods

n/a	Involved in the study	
☒	☐	ChIP-seq
☒	☐	Flow cytometry
☒	☐	MRI-based neuroimaging

Eukaryotic cell lines

Policy information about cell lines and Sex and Gender in Research

Cell line source(s)	Human embryonic kidney (HEK) 293 cells (Catalog Number: GNHu43) provided by the Shanghai Institute of Biochemistry and Cell Biology, China.
Authentication	Cell line is authenticated with short tandem repeat test.

Mycoplasma contamination	The cell line is tested negative for mycoplasma contamination.
Commonly misidentified lines (See ICLAC register)	No commonly misidentified line is used in this study.

Animals and other research organisms

Policy information about studies involving animals; ARRIVE guidelines recommended for reporting animal research, and Sex and Gender in Research

Laboratory animals	7 weeks old female Balb/cA mice
Wild animals	Not applicable.
Reporting on sex	Sex is not considered in study design because this study is to develop temperature imaging method with high spatial resolution and used cerebral vessels of mice to demonstrate the imaging spatial resolution, which did not study the possible biological difference among different sexes of animal model.
Field-collected samples	The study did not involve the samples collected from the field.
Ethics oversight	The animal procedures were approved by the Institutional Animal Care and Use Committee, ShanghaiTech University.

Note that full information on the approval of the study protocol must also be provided in the manuscript.

Plants

Seed stocks	Not applicable.
Novel plant genotypes	Not applicable.
Authentication	Not applicable.
